# Identifying binary protein-protein interactions from affinity purification mass spectrometry data

**DOI:** 10.1186/s12864-015-1944-z

**Published:** 2015-10-05

**Authors:** Xiao-Fei Zhang, Le Ou-Yang, Xiaohua Hu, Dao-Qing Dai

**Affiliations:** School of Mathematics and Statistics, Central China Normal University, Luoyu Road, Wuhan, 430079 China; Intelligent Data Center and Department of Mathematics, Sun Yat-Sen University, Xingang West Road, Guangzhou, 510275 China; School of Computer, Central China Normal University, 774 Luoyu Road, Wuhan, 430079 China; College of Information Science and Technology, Drexel University, Chestnut Street, Philadelphia, 19104 USA

**Keywords:** Protein-protein interactions, Direct physical interactions, Scoring methods, Affinity purification mass spectrometry data

## Abstract

**Background:**

The identification of protein-protein interactions contributes greatly to the understanding of functional organization within cells. With the development of affinity purification-mass spectrometry (AP-MS) techniques, several computational scoring methods have been proposed to detect protein interactions from AP-MS data. However, most of the current methods focus on the detection of co-complex interactions and do not discriminate between direct physical interactions and indirect interactions. Consequently, less is known about the precise physical wiring diagram within cells.

**Results:**

In this paper, we develop a Binary Interaction Network Model (BINM) to computationally identify direct physical interactions from co-complex interactions which can be inferred from purification data using previous scoring methods. This model provides a mathematical framework for capturing topological relationships between direct physical interactions and observed co-complex interactions. It reassigns a confidence score to each observed interaction to indicate its propensity to be a direct physical interaction. Then observed interactions with high confidence scores are predicted as direct physical interactions. We run our model on two yeast co-complex interaction networks which are constructed by two different scoring methods on a same combined AP-MS data. The direct physical interactions identified by various methods are comprehensively benchmarked against different reference sets that provide both direct and indirect evidence for physical contacts. Experiment results show that our model has a competitive performance over the state-of-the-art methods.

**Conclusions:**

According to the results obtained in this study, BINM is a powerful scoring method that can solely use network topology to predict direct physical interactions from AP-MS data. This study provides us an alternative approach to explore the information inherent in AP-MS data. The software can be downloaded from https://github.com/Zhangxf-ccnu/BINM.

**Electronic supplementary material:**

The online version of this article (doi:10.1186/s12864-015-1944-z) contains supplementary material, which is available to authorized users.

## Background

Proteins often perform their functions through physically binding with other partners. Thus, the identification of direct physical protein-protein interactions is critical in elucidating the structural and functional architecture of the cell [1], and further in exploring mechanisms of human diseases [2].

There are two leading high throughput experimental technologies for identifying protein interactions – yeast two-hybrid (Y2H) [3–5] and affinity purification followed by mass spectrometry (AP-MS) [6–8]. Y2H screening is a widely used technique to discover direct physical interactions between proteins. Due to protocol-specific biases, interactions identified by Y2H are enriched with transient, condition-specific and inter-complex interactions [9] and have high levels of false positives and false negatives [10, 11]. Therefore, Y2H is not sufficient to obtain a precise and comprehensive binary interaction map within protein complexes and cells.

AP-MS is an alternative approach to detect interactions within protein complexes. Unlike Y2H that focuses on detecting direct physical interactions, AP-MS is designed to identify co-complex interactions which include both direct physical interactions (between proteins that share a common binding interface) and indirect co-complex associations (between proteins that do not physically interact with each other, but belong to common complexes) [12–14]. Recently, several scoring methods have been proposed to predict co-complex relationships from AP-MS data [6, 7, 13, 15–19]. However, most of them do not distinguish between direct interactions and indirect interactions [13]. Consequently, less is known about the internal topology and the physical interactions within the protein complexes [5, 13, 20]. To address this problem, we attempt to predict direct physical interactions from AP-MS data by discriminate between direct interactions and indirect interactions. For the sake of convenience, we use “direct interaction”, “physical interaction” and “binary interaction” interchangeably in the rest of the text.

Although AP-MS is not designed to identify binary interactions, based on the data it generates, we could distinguish direct interactions from indirect interactions by analyzing the topological structure of AP-MS data [12, 20–22]. The spoke and matrix models are two classical approaches to transform co-complex associations into direct interactions. The spoke model considers only bait-prey interactions; while the matrix model takes into account both bait-prey and prey-prey interactions. In general, the spoke model has a high false negative rate; while the matrix model has a high false positive rate [21]. Friedel and Zimmer [20] presented a method for identifying direct interactions by calculating the union of all maximum spanning trees (MST) of a given co-complex network. Their method strictly classified co-complex interactions into direct physical interactions and indirect co-complex interactions, but did not reassign scores to interactions to quantify their reliability. Therefore, the performance may depend heavily on the accuracy of data under consideration. Kim et al. [12] proposed a new method to distinguish direct interactions from indirect ones. However, it is computational expensive [12]. Recently, Saraç et al. [22] developed a supervised classification-based method to distinguish binary, co-complex and functional interactions in functional networks. However, they did not investigate the performance of their method on detecting direct interactions from AP-MS data.

In gene regulatory network inference literature, two landmark methods have been proposed to cleanup the observed network which includes direct links obscured by indirect links [23, 24]. Both methods are based on two assumptions: first, the observed network is the sum of both direct and indirect links; second, an indirect link from a source to a target is mediated by the direct neighbors of the target. They developed different mathematical methods to derive the strengths of direct links. Links with large strengths are kept as direct links; whereas links with small strengths are treated as indirect ones and removed.

Inspired by the two prominent methods, we develop a Binary Interaction Network Model (BINM) to discriminate the direct and indirect interactions in co-complex interaction network. BINM introduces a parameter to represent the likelihood that an observed co-complex interaction would be direct. It assumes that the observed co-complex interactions are the sum of both direct and indirect interactions, and that indirect interactions are captured by direct interactions through the common neighbors. Based on these two assumptions, BINM can well capture the relationships between observed co-complex interactions and the underlying direct interactions. In particular, for an observed co-complex interaction network which is inferred from AP-MS data, it identifies direct physical interactions using the estimators of model parameters. We test our model on two yeast datasets which are constructed from a same combined purification data using two different scoring methods. The performance is assessed using different types of reference sets that represent complementary evidence for physical interactions: (1) reference sets that derived from available direct physical interactions, (2) reference sets that derived from three-dimensional structural information, (3) reference sets that derived from manually curated protein complexes, and (4) reference sets that derived from genetic interaction profiles. Comparative experiments demonstrate that our model has a better performance than state-of-the-art methods.

## Methods

### AP-MS data sets

We use a combined set of purifications from two independent large-scale screens in Saccharomyces cerevisiae [6, 7]. Two scoring methods [15, 16] are used to identify high confidence co-complex interactions from the combined AP-MS dataset. Collins et al. [15] used a scoring scheme called purification enrichment (PE) to analyze the combined purification data. Applying a score threshold (of 3.19), they identified 9070 high confidence interactions among 1622 proteins. They also used LOESS regression [25] and the pool adjacent violators algorithm [26] to scale the PE scores onto the interval 0−1. Here we focus on the 9070 high confidence interactions and use the scaled scores to represent their reliability. These interactions and their scores can be downloaded from the supporting web-site (http://interactome-cmp.ucsf.edu/). Friedel et al. [16] used the bootstrap technique [27, 28] to determine confidence scores. They calculated Bootstrap confidence scores for 62876 interactions between 5195 proteins. All Bootstrap confidence scores are between 0 and 1. Here we only consider interactions with confidence scores ≥0.1, and we obtain 10096 interactions between 2684 proteins. We use a cutoff of 0.1 such that only about 10000 high confidence interactions are considered. Note that although there exists direct interactions beyond the cutoff of 0.1, interactions at this low cutoff have only very low confidence and are thus omitted [13]. The Bootstrap scores for the combined purification data can be downloaded from http://www.bio.ifi.lmu.de/Complexes. We refer to the two datasets as Collins and Friedel, respectively. The two datasets are constructed using different scoring schemes on a same combined set of purification observations, therefore they can be used to test how different AP-MS scoring schemes affect the performance of the proposed model. The detailed description of the statistical model used to derive the types of confidence scores is beyond the scope of the paper and the interested reader is referred to the original publications [15, 16].

### Reference data sets

Because no true gold standard for direct physical interactions is available, we compile three independent and complementary reference sets of binary interactions. We first use the entire high-quality binary interactions in the HINT database (version 6/17/2015) as a gold standard [29]. The HINT database collected interactions from several databases and filtered them to remove low-quality interactions. Therefore, this reference set has a high reliability and coverage. In a similar manner to [13, 20], we also compile two reference sets using all interactions determined with yeast two-hybrid assays (denoted as Y2H) [4] and protein-fragment complementation assay (denoted as PCA) [30] in the BioGRID database (version 3.4.125) [31]. The three reference sets provide direct evidence for physical interactions.

We also construct a reference set to estimate the quality of the inferred direct interactions on the level of three-dimensional structure. Zhang et al. [32] calculated a likelihood ratio through structural modeling for each pair of proteins to indicate whether they interact physically. Their study showed the effect of three-dimensional structural information on the identification of physical interactions. Therefore, the three-dimensional structure information-based score is an effective data to test the performance of our model. The structure-derived scores are obtained from the PrePPI database [33].

We use the CYC2008 [34] and SGD [35] benchmarks as the gold standards of yeast protein complexes. The CYC2008 catalogue is downloaded from http://wodaklab.org/cyc2008/ on July 4, 2015. We derive the SGD complexes following the methods of [36, 37]. The SGD annotations and the cellular component ontology used to generate the SGD complexes are downloaded from the Gene Ontology database (version: July 4, 2015) [38]. GO annotations with the IEA, ND, NAS evidences and the NOT qualifier are not considered here.

The genetic interaction profiles are obtained from a recent large-scale functional study in yeast [39]. We download the lenient cutoff interaction set from http://drygin.ccbr.utoronto.ca/~costanzo2009/. We focus on the lenient cutoff dataset because it offers the highest covered proteins and includes only statistically significant interactions. Then we compute the genetic profile similarities for pairs of proteins by computing Pearson’s correlation coefficients between their the genetic interaction profiles. Analogous to [13, 39], all protein pairs with a genetic interaction profile similarities ≥0.2 are used to construct a functional map. Protein pairs in this map define a genetic interaction reference set.

### Binary interaction network model

**Fig. 1:**
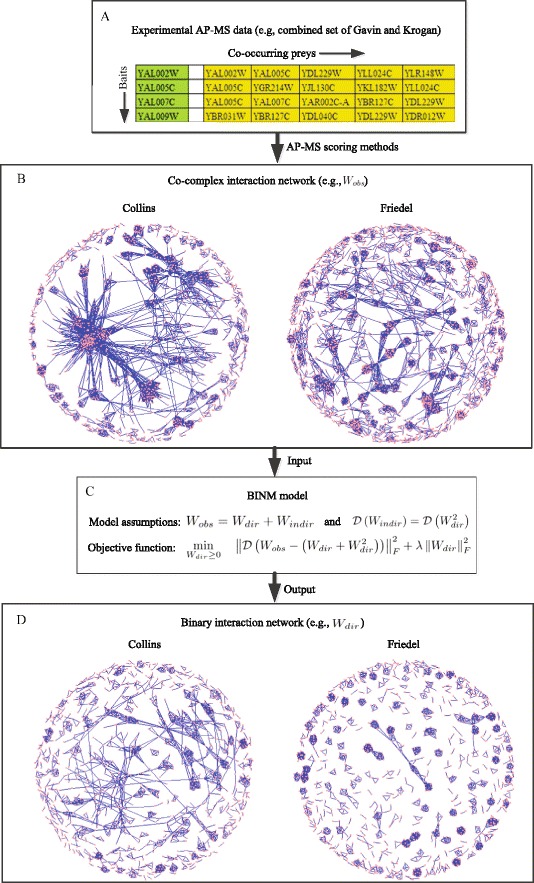
Flow chart of binary interaction network model. **a** Experimental AP-MS data. It includes two type of observations: bait-prey observations and prey-prey observations. **b** Co-complex interaction network (*W*_*obs*_). It can be derived from AP-MS data using some scoring schemes. It often contains both direct physical interactions and indirect interactions with no clear separation. This map illustrates the Collins and Friedel datasets. **c** Schematic overview of BINM model. Our model captures topological relationships between direct physical interactions and observed co-complex interactions in terms of the following assumptions: 1) the observed co-complex interactions are the sum of both direct physical interactions and indirect interactions; 2) the indirect interactions are captured by direct interactions through the common neighbors. Then the binary interaction detection problem can be transformed into a nonnegative constrained optimization problem. **d** Binary interaction network (*W*_*dir*_) identified by BINM. After finding an optimal solution for the BINM model, the observed interactions are ranked by their BINM scores (e.g., *W*_*dir*_). Top ranked interactions can be predicted as direct physical interactions. The map depicts binary networks induced by the top 2562 and 3018 interactions for the Collins and Friedel datasets, respectively. We discuss how to determine the rank cutoffs in the Results section

Before introducing our model, we first give some notations and formalize the problem. Given a set of observed weighted co-complex interactions between *n* proteins, we use a weighted network with adjacency matrix *W*_*obs*_ to model it. The matrix *W*_*obs*_ stores the confidence scores of these interactions, where each entry $w^{obs}_{\textit {ij}}$ represents the likelihood that proteins *i* and *j* belongs to a common complex. Interactions not observed are given a weight of 0. We do not consider self-interactions in this study. This matrix can be derived from AP-MS data using some scoring methods (Fig. 1a-b). We assume that all confidence scores are in the range of 0 to 1. If the scores are from −*∞* (or 0) to *∞*, they should be scaled to [0,1].

Since the co-complex interaction network derived from AP-MS data contain both direct physical interactions and indirect interactions (Fig. 1b), the binary interactions cannot be derived from *W*_*obs*_ directly. To this end, two nonnegative matrices $W_{\textit {dir}} = \left (w^{dir}_{\textit {ij}} \right)$ and $W_{\textit {indir}} = \left (w^{indir}_{\textit {ij}} \right)$, which are initially unknown, are introduced to represent the strengths of direct and indirect interactions, respectively. The problem of identifying binary interactions from AP-MS data is then converted to the estimation of *W*_*dir*_ given *W*_*obs*_. Once the estimator of *W*_*dir*_ is obtained, we can predict binary interactions according to the value of this estimator (a higher value indicates the corresponding two proteins are more likely to physically interact with each other).

Now we introduce a mathematical model, named as binary interaction network model (BINM), to capture the topological relationship between observed co-complex interaction matrix *W*_*obs*_ and direct interaction matrix *W*_*dir*_. The observed co-complex interaction confidence scores are usually constructed according to two types of observations: direct bait-prey observations and indirect prey-prey observations where two proteins are both identified as preys in a purification with a same protein as bait [6, 15, 16]. Therefore, we intuitively assume that the observed interactions are the sum of direct physical interactions and indirect interactions; that is

(1) $$ W_{obs} = W_{dir} + W_{indir}.  $$

The indirect co-complex association score between two proteins *i* and *j* is usually calculated based on the co-occurrence of them as preys in the same purifications. The more common bait proteins two prey proteins share, the more likely they belong to same complexes. Therefore, we assume the confidence score of indirect interaction between proteins *i* and *j* (*i*≠*j*), $w^{indir}_{\textit {ij}}$, can be captured by summing the effects mediated by the common neighbors of them in binary interaction network *W*_*dir*_, that is $w^{indir}_{\textit {ij}} = \sum _{k=1}^{n} w^{dir}_{\textit {ik}}w^{dir}_{\textit {kj}}$. The above relationship can be rewritten in matrix form as follows:

(2) $$ \mathcal{D} \left(W_{indir} \right)= \mathcal{D} \left(W_{dir}^{2}\right).  $$

Here $\mathcal {D}(\cdot)$ sets the diagonal terms of a matrix to zero such that self-interactions are omitted.

For an observed co-complex interaction network *W*_*obs*_, the question is how to estimate *W*_*dir*_ according to Eqs. (1) and (2). Instead of solving the non-linear equations, we transform this problem into the following optimization problem by taking Eq. (2) into Eq. (1) (Fig. 1c):

(3) $$  \min_{W_{dir} \ge 0} \quad \left\|\mathcal{D} \left(W_{obs} - \left(W_{dir}+ W_{dir}^{2}\right) \right) \right\|_{F}^{2} + \lambda \left\|W_{dir}\right\|_{F}^{2},  $$

where ∥·∥_*F*_ denotes the Frobenius norm of a matrix, and *W*_*dir*_≥0 means each entry $w^{dir}_{\textit {ij}} \ge 0$. The first term represents the error which quantifies how well the observed co-complex interactions can be captured by the direct physical interactions in terms of quadratic loss function. Since the observed co-complex interactions have high levels of noise [11], a complex estimator of *W*_*dir*_ that approximates the observed data best may result in overfitting. Therefore, we introduce a regularization term, $\left \|W_{\textit {dir}}\right \|_{F}^{2}$ (the second term), to penalize complex estimators in a similar manner to ridge regression [25]. Here *λ*≥0 is a tuning parameter that balances the two terms. Selecting a good value of *λ* is critical; we will discuss the effect and choice of *λ* in the next section.

To optimize Eq. (3) for *W*_*dir*_, we adopt the multiplicative update rule [40] which is a special case of gradient descend method that keeps nonnegativity of *W*_*dir*_ through an automatic step selection. According to this rule, we obtain the following updating formula:

(4) $$ \begin{aligned} W_{dir} \leftarrow W_{dir} \cdot \left(\frac{W_{obs} + W_{dir}{{\!~\!}^{T}} W_{obs} + W_{obs}W_{dir}{{\!~\!}^{T}}} {\hat{W}_{obs} + W_{dir}{{\!~\!}^{T}} \hat{W}_{obs} + \hat{W}_{obs} W_{dir}{{\!~\!}^{T}} + \lambda W_{dir}} \right){\!~\!}^{. \frac{1}{4}}, \end{aligned}  $$

where $\hat {W}_{\textit {obs}}$ is computed using $\hat {W}_{\textit {obs}} = \mathcal {D}\left (W_{\textit {dir}} + W_{\textit {dir}}^{2} \right)$. That is, $\hat {W}_{\textit {obs}}$ is an approximation of *W*_*obs*_ based on current estimator of *W*_*dir*_, and the diagonal terms of it are set to zero. Here matrix operation *X*·*Y* represents element-by-element multiplication; $\frac {X}{Y}$ represents element-by-element division; and $X^{. \frac {1}{4}}$ represents element power. Due to the lack of space, the details of this updating rule are presented in Additional file 1.

The procedure of identifying binary interactions from co-complex interaction network using BINM is presented in Algorithm 1. We set the diagonal terms of *W*_*obs*_ to zeros such that self-interactions are omitted. In the iteration process, we initialize *W*_*dir*_ using *W*_*obs*_. In this way, according to update formula (4), the estimator $w^{dir}_{\textit {ij}}$ will be 0 if ${w}^{obs}_{\textit {ij}} = 0$. Therefore, our model is developed to reassign weights to observed co-complex interactions to quantify the strengths of direct physical interactions, but not to predict new interactions. The consecutive update of *W*_*dir*_ is conducted until the relative change in the objective value of Eq. (3) is less that 0.1 *%*. To avoid the case that this process converges too slowly, we also stop it if the number of iterations reaches 20. After estimating *W*_*dir*_, we can rank these observed interactions according to the magnitude of ${w}^{dir}_{\textit {ij}}$. The top-ranked interactions can be predicted as direct physical interactions (Fig. 1d). In this paper, we refer to *W*_*dir*_ as BINM scores.



## Results

In this section, we first analyze the effect of parameter. Then, we assess the ability of various methods in detecting direct physical interactions from AP-MS data. Because there is no comprehensive gold standard set of physical interactions [13], we assess the performance of various methods from different perspectives. First, we drive three complement reference sets of binary interactions, and compare the top-ranked interactions to these reference sets. Since these reference sets represent only a small fraction of direct physical interactions, we also resort to several additional reference sets that are derived from three-dimensional structural information of protein interactions, manually curated protein complexes, and genetic interaction profiles. We also analyze the difference between score distributions of bait-prey interactions and prey-prey interactions. Because spoke model is often considered to be better suited to detect direct physical interactions, we compare our model with a spoke model. Finally, binary interaction networks are constructed by a simple thresholding method.

### Effect and determination of parameter

There is a parameter *λ* in the proposed BINM model. We wonder how it influences the performance. We run the BINM model on the two co-complex interaction networks (Collins and Friedel) with different values of *λ* (*λ*∈{2^−4^,2^−3^,⋯,2^2^}), and evaluate the performance using the three reference sets of direct physical interactions (HINT, Y2H and PCA). The performance is measured by the area under the receiver operating characteristic curve (AUC), which is equal to the probability that a method will rank a randomly chosen direct physical interaction (positive instance) higher than a randomly chosen indirect co-complex association (negative instance) [41]. Larger scores of AUC are better.

**Fig. 2:**
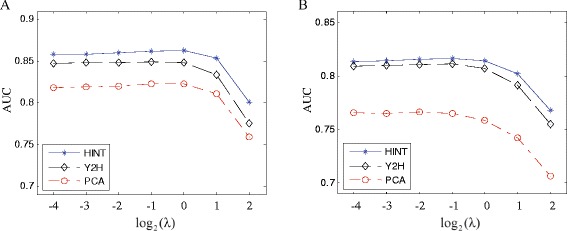
Performance of our model with different values of *λ* measured by AUC scores with respect to reference sets of direct interactions. The x-axis denotes the value of log2*λ*; the y-axis denotes the value of AUC score. **a** Collins dataset, **b** Friedel dataset

Figure 2 shows the performance of our model with respect to various values of *λ*. As the value of *λ* increases, the AUC scores increases slightly in the beginning and decreases after obtaining maximum. Overall, the AUC scores do not change significantly when *λ*∈[2^−4^,1], which shows that BINM is not very sensitive to the choice of *λ*. To avoid overestimating the performance of our model, we do not tune the parameter to a particular dataset and set *λ* to 1 as the default value in the following experiments.

### Performance evaluation using direct physical interactions as reference sets

To investigate the predictive accuracy, three reference sets of experimentally validated binary protein interactions are complied: HINT, Y2H and PCA. We compare BINM with AP-MS [15, 16], MST and eMST [20]. For the method of AP-MS, the performance is evaluated using the co-complex confidence scores (*W*_*obs*_) which are calculated using some scoring methods. For the collins dataset, confidence scores are calculated using the PE scoring method [15]; for the Friedel dataset, the scores are calculated using a bootstrap method [16]. Please note that AP-MS is different from the other three methods (BINM, MST and eMST) because that the AP-MS scores are calculated using the raw purification data while the other three methods make predictions based on the AP-MS scores. In a similar manner to [23, 24], the AP-MS scoring methods are used as a baseline to test whether BINM is able to improve the discrimination power between direct interactions and indirect interactions. The softwares of MST and eMST are downloaded from http://www.bio.ifi.lmu.de/Complexes/Substructures/. For eMST, we use the default parameter *α*=1. The method of [12] is not considered since there is no public software available and it is computational expensive. The predictions made by [22] are not evaluated for the following reasons. First, unlike the unsupervised methods we consider, their method is supervised. The evaluation experiments can only be implemented by cross-validation which may lead to biases and overestimation. Second, they focus on classifying interactions in functional interaction networks, while we pay attention to distinguish direct physical interactions and indirect interactions in co-complex interaction network which is inferred from AP-MS data.

**Fig. 3:**
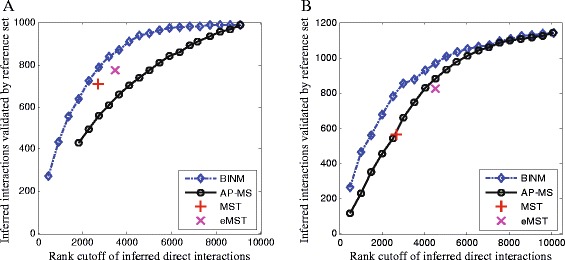
Assessment of inferred direct interactions against the HINT reference set. Interactions are ranked by scores calculated using corresponding methods. Performance of all methods is measured by plotting the number of top-ranking inferred direct interactions of a method against the number of these interactions that are validated by the HINT reference set. **a** Collins dataset, **b** Friedel dataset

Figure 3 and Figures S1-S2 in Additional file 1 show how well different methods perform in identifying direct interactions in terms of the three reference sets. The performances of MST and eMST are data dependent. They outperform AP-MS on the Collins dataset, but eMST works a little poorer than AP-MS on the Friedel dataset when comparing against the HINT and Y2H reference sets. Notable, BINM outperforms the other three methods on the two datasets with respect to all the three reference sets. These results show that BINM is less sensitive to the scoring methods, which are used to construct co-complex interaction network from AP-MS data, than MST and eMST. We also evaluate the performance using the receiver operating characteristic (ROC) curve. The results also show that BINM performs better than other competing methods (Figures S3-S5 in Additional file 1).

### Performance evaluation using three-dimensional structural information

Owing to the fact that the reference sets of binary interactions are incomplete, a predicted direct physical interaction that does not belong to the reference sets may be a valid but previous uncharacterized binary interaction. Therefore, we conduct a complementary experiment to assess the reliability of the predicted direct interactions using the three-dimensional structural information. This is inspired by the fact that three-dimensional structural information can be used to predict physical interactions with considerable accuracy [32]. Here we use the structure-based scores provided by [33] which quantify the likelihood ratio (LR) that a candidate pair of proteins represents a true direct physical interaction.

**Fig. 4:**
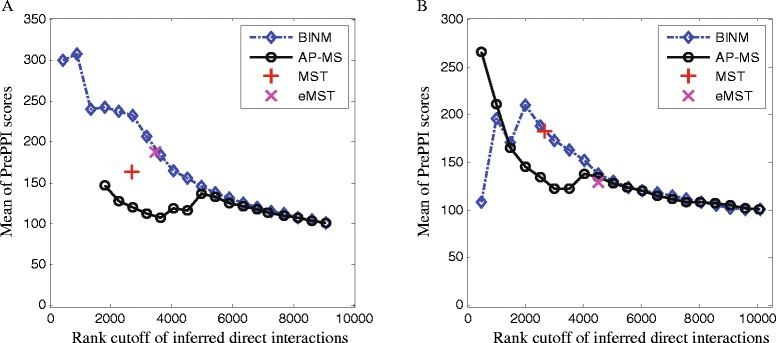
Assessment of inferred direct interactions using the three-dimensional structural information. Interactions are ranked by scores calculated using corresponding methods. Performance of all methods is measured by plotting the number of top-ranking inferred direct interactions of a method against the mean of PrePPI scores of these interactions. **a** Collins dataset, **b** Friedel dataset

We rank interactions according to their confidence scores derived from different methods, and we compute the average likelihood ratios of the corresponding pair-wise interactions. Please note that we do not consider interactions which are not assigned with structural scores (e.g., LR =0). Figure 4 shows the results of the aforementioned four methods. At a same rank cutoff, the structural scores of interactions inferred by BINM are consistently higher than the scores of interactions inferred by the three compared methods (except the top 1,500 predictions on the Friedel dataset).

### Performance evaluation using protein complexes

A protein complex is a group of proteins that physically interact with each other to fulfill their functions [36, 42]. Therefore, we can rely on the following assumption to assess the identified direct physical interactions: all members of a protein complex can be connected by the physical interactions [13]. Consequently, the quality of inferred physical interactions can be estimated by assessing how well these physical interactions connect the member proteins of manually curated protein complexes. Here we use the SGD and CYC2008 complexes to assess the performance.

**Fig. 5:**
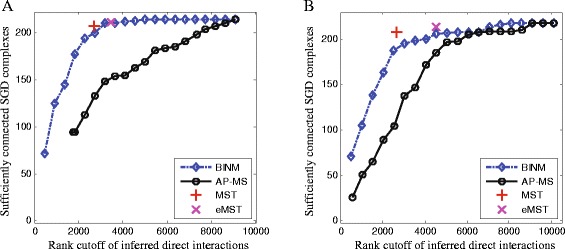
Assessment of inferred direct interactions using the SGD complexes. Interactions are ranked by scores calculated using corresponding methods. Performance of all methods is measured by plotting the number of top-ranking inferred direct interactions of a method against the number of complexes that are sufficiently connected by these interactions. A complex is considered to be sufficiently connected by a set of physical interactions if the physical interactions reduce the number of connected components within the complex to less than 50 *%* compared with the unconnected complex. **a** Collins dataset, **b** Friedel dataset

Figure 5 and Figure S6 in Additional file 1 depict how well physical interactions predicted by different methods connect manually curated protein complexes. Following in the method of [13], a complex is considered to be sufficiently connected by a set of inferred physical interactions if these interactions reduce the number of connected components within the complex to less than 50 *%* compared with the unconnected complex. It is noticeable that the physical interactions identified by BINM, MST and eMST can sufficiently connect more complexes than those identified by AP-MS at a same rank cutoff. We also observe that MST and eMST seem to be a little better suited to connect complexes than BINM. This might be due to the fact that MST and eMST identify physical interactions on the basis of maximum spanning trees which take into account how well the inferred direct interactions connect the entire network.

### Performance evaluation using genetic interaction profiles

**Fig. 6:**
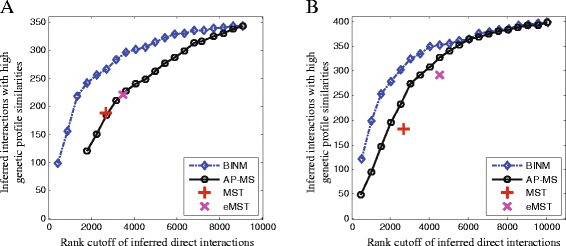
Assessment of inferred direct interactions using the genetic interaction profiles. Interactions are ranked by scores calculated using corresponding methods. Performance of all methods is measured by plotting the number of top-ranking inferred direct interactions of a method against the number of these interactions which also have high genetic profile similarities. Functional similarity of proteins is measured by Pearson’s correlation between their genetic interaction profiles, and protein pairs with functional similarities ≥0.2 are used to construct the genetic interaction reference set. **a** Collins dataset, **b** Friedel dataset

Physically interacting proteins that carry out similar biological functions often have strongly correlated genetic interaction profiles [39]. Therefore, we can use genetic interaction profiles as another evidence to assess the identified physical interactions. Following the method of [13], we obtain a set of genetic interaction profiles from [39] and employ it to define a genetic interaction reference set which consists of protein pairs with high genetic interaction profile similarities. Figure 6 illustrates how well physical interactions inferred by different methods match with the genetic interaction reference set. This assessment shows that BINM significantly outperforms the other three methods.

### Score distributions of different types of interactions

AP-MS experiments identify protein interactions using both a bait protein and a set of prey proteins. Bait-prey (B-P) observations provide evidence for direct physical interactions, and prey-prey observations provide evidence for indirect co-complex associations [15]. Therefore, it would be interest to test whether the confidence scores (e.g., *W*_*obs*_ and *W*_*dir*_) can distinguish bait-prey interactions and prey-prey interactions. We obtain the raw purification data from http://interactome-cmp.ucsf.edu/. A protein pair is considered to be a bait-prey interaction if one of them is a bait protein; otherwise, it is considered to be a prey-prey interaction.

**Fig. 7:**
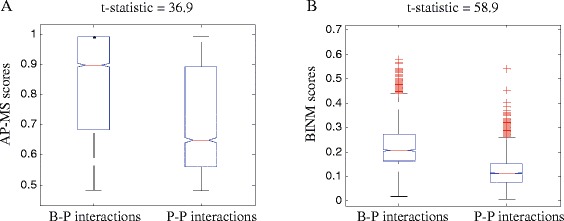
Score distributions of different types of interactions on the Collins dataset. The score distributions are represented by box plots (line = median). We use Student’s t-test to test difference between scores of bait-prey (B-P) interactions and prey-prey (P-P) interactions, and t-statistics is presented in the figure. **a** AP-MS score distributions, **b** BINM score distributions

From Fig. 7 and Figure S7 in Additional file 1, we observe the confidence scores of bait-prey interactions are, on average, higher than those of prey-prey interactions for both AP-MS and BINM scoring methods. The statistical significance of theses differences is validated using Student’s t-test (P-value ≤0.001). Furthermore, we find that the t-statistics of BINM scores are higher than those of AP-MS scores. These results show that, compared with the AP-MS scoring method, our model can increase the discrimination power between bait-prey interactions and prey-prey interactions.

### Comparison with spoke model

The computational methods that assign confidence scores to AP-MS observations belong to two major categories: methods that only consider bait-prey observations (spoke model) and those that consider both bait-prey and prey-prey observations (matrix model) [19]. The spoke model may be better suited to identify direct physical interactions from AP-MS data than the matrix model [13]. In this study, we first use two scoring methods which is based on matrix model to infer the co-complex interaction network from AP-MS data, and then use BINM to identify direct physical interactions from the co-complex interactions. Therefore, it is interesting to compare our model with spoke model which can directly identify physical interactions from AP-MS data.

**Fig. 8:**
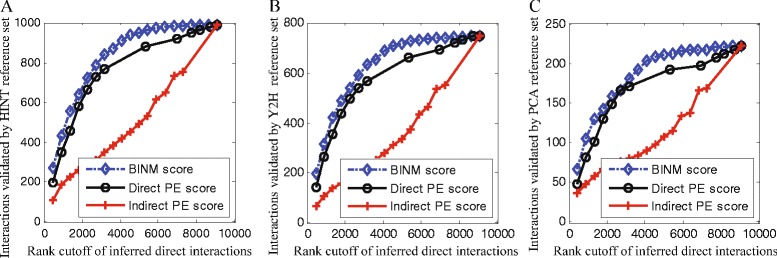
Assessment of BINM, direct PE and indirect PE scores on the Collins dataset. Interactions are ranked by corresponding scores. Performance of all methods is measured by plotting the number of top-ranking inferred direct interactions against the number of these interactions that are validated by reference sets. **a** HINT reference set, **b** Y2H reference set, **c** PCA reference set

Collins et al. [15] calculated PE scores as a sum of direct bait-prey components (denoted as direct PE scores) and indirect prey-prey components (denoted as indirect PE scores). We compare BINM scores (e.g., *W*_*dir*_) with direct PE scores and indirect PE scores to test how well they perform in identifying direct physical interactions. Direct PE scores and indirect PE scores are obtained from the supporting web-site. The performance is assessed using the three reference sets of binary interactions: HINT, Y2H and PCA. As can be seen from Fig. 8, BINM achieves the best performance among all the three scoring methods and direct PE scoring scheme significantly outperforms indirect PE scoring scheme. These results show that prey-prey observations mainly provide evidence for indirect co-complex interactions and are not well suited to infer direct physical interactions. However, BINM can effectively combine prey-prey observations with bait-prey observations to improve the performance. Here we do not consider the Friedel dataset since Friedel et al. [16] did not provide scores of the two types of observations separately.

### Binary interaction network

To construct a comprehensive binary interaction network, we rank the observed co-complex interactions according to their BINM scores and predict the top-ranked interactions as binary interactions. Here, determining a reasonable rank cutoff is a critical problem since a low cutoff will produce a small network with low coverage (recall), and a high cutoff will produce a large network with low reliability (precision). We need to pick up an optimal cutoff that has a good compromise between coverage and reliability. To this end, we try different rank cutoffs and compare the resulting binary networks against the HINT reference set using the *F*_2_ measure [43, 44]. We use the HINT reference set because it is high quality. Unlike the traditional *F*_1_ measure which weights recall and precision equally, *F*_2_ measure puts higher weights on recall than precision. Here we use *F*_2_ measure because that the reference set of binary interactions is not complete and recall may be more important than precision.

**Fig. 9:**
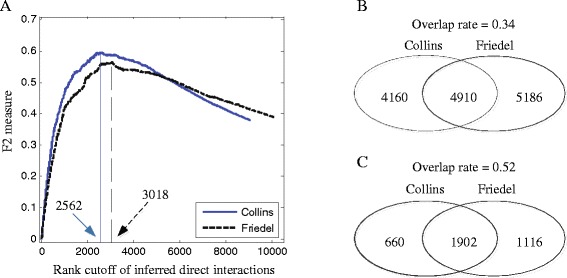
Induced binary interaction network. **a** The method of determining cutoff. Interactions are ranked by their BINM scores. Different values of cutoff are used to predict binary interactions, and the corresponding *F*_2_ scores are calculated using these predictions. Then we use the value that produces the largest *F*_2_ score to construct binary network. For the Collins dataset, we use a cutoff of 2562; for the Friedel dataset, we use a cutoff of 3018. **b** Overlap between co-complex interactions in the Collins and Friedel datasets. **c** Overlap between the direct physical interactions identified by BINM from the Collins and Friedel datasets

Figure 9a depicts the method of determining the rank cutoffs. We observe that as the cutoff increases, the *F*_2_ score increases in the beginning and then decreases after obtaining its maximum. This is because that if the cutoff is too low, only a small fraction of direct physical interactions can be detected and we will obtain a low recall; while if the cutoff is too high, a large fraction of indirect interactions will be predicted as direct interactions and we will obtain a low precision. Rank cutoffs of 2562 and 3018 produce the highest *F*_2_ scores for the Collins and Friedel datasets, respectively. We then use these two cutoffs to construct the binary interaction networks (Fig. 1d). The inferred binary interaction networks are sparse and modular, which agree with our intuition for the networks of direct physical interactions with protein complexes [13]. Since the Collins and Friedel datasets are inferred from a same AP-MS data, it is interesting to assess the agreement between the interactions generated by different methods. There are 4910 interactions shared by the two datasets which are inferred using the PE and bootstrap scoring methods respectively (Fig. 9b). Furthermore, there are 1902 interactions that are in common between the binary interactions identified by our model from the two datasets (Fig. 9c). The overlap rate (Jaccard index) between interactions in the two datasets can be increased from 0.34 to 0.52 through using our model to filter out indirect interactions.

## Discussion

Different from Y2H screening that discovers binary interactions, AP-MS identifies co-complex interactions between proteins. Therefore, direct physical interactions in AP-MS data are obscured by indirect co-complex interactions. In this paper, we have presented a new network topology-based method to identify binary interactions in co-complex interaction network which is induced from AP-MS data. Unlike the transient and inter-complex binary interactions discovered by Y2H, the binary interactions identified by our method from AP-MS data are enriched with stable and intra-complex interactions. The two types of interactions are fundamentally different and complementary, which indicates that our predictions can substantially expand the knowledge about pairwise binary interactions.

Previous AP-MS scoring methods focus on assigning confidence scores that represent strength of co-complex relationships to interactions observed within the purifications. Therefore, a high confidence score between two proteins only indicate that they are likely to belong to a common complex but can not give clues to whether or not they interact physically. Consequently, direct physical interactions and indirect co-complex interactions can not be separated simply. Here, we resort to the method of network deconvolution [23, 24] and reassign confidence scores that represent strength of direct interactions to co-complex interactions identified using previous scoring methods. Specially, our model enhances the scores of direct interactions and silences the scores of indirect interactions. By doing so, direct interactions and indirect interactions can be simply separated using a thresholding method.

BINM is a post-processing scoring scheme that is devised to identify binary interactions from co-complex interactions. Interactions with BINM scores exceeds a predefined threshold can be predicted as direct interactions. In comparative experiments, we rank interactions according to their scores and use different rank cutoffs to make predictions. Different methods are then compared at a same cutoff. For practical application purpose, determining a reasonable cutoff is a critical problem since different cutoffs will produce networks with different reliability and coverage. Based on a given gold standard of direct interactions, we can pick up a cutoff that produces the highest *F*_2_ score. However, this method depends on a complete gold standard set of binary interactions which is not available at present. It might be possible to follow the method of false discovery rate (FDR) [18, 19]. We do not make further efforts to this method because how to calculate FDR and determine a reasonable FDR threshold have many open problems themselves.

Before using our model, we need to construct a co-complex interaction network from AP-MS data using a predetermined scoring scheme (Fig. 1a-b). Therefore, the performance of our model may be influenced by the the scoring scheme we use. To assess the influence of scoring schemes, we derive two co-complex interaction networks from a same combined AP-MS data but using different scoring schemes. Experiment results show that our model outperform competing methods on both datasets, which demonstrate that the performance of our model is not very sensitive to the scoring schemes. In practise, we suggest using matrix model-based scoring schemes to infer weighted co-complex interactions. This is because spoke model consider only bait-prey interactions where physical interactions between prey proteins may be overlooked. Take the Friedel dataset and HINT reference set for example, there are about 100 out of 1144 direct physical interactions between prey proteins. Furthermore, we have shown that our model outperforms the spoke model when using the PE scoring scheme. Therefore, besides bait-prey observations, prey-prey observations are also useful to identify binary interactions.

The proposed method has a number of desirable properties. First, it is effective and simple. Compared to the method of [20], the superior performance of our method has been shown in the experiments. Unlike the supervised classification method developed in [22], our method is unsupervised. Defining positive and negative examples, which itself has many open problems [45], is not necessary. Second, our method is based on well established theories of matrix approximation and regularization which have sound mathematical principles. It may be more preferable over methods based on a mere hunch. Third, it is fast and scale well with the size of the network analyzed. As discussed in Additional file 1, the worst time cost of our algorithm is *O*(*nTE*), where *n* is the number of proteins, *E* is the number of interactions and *T* is the number of iterations. We implement the algorithm using Matlab in a workstation with Intel 4 CPU (3.40 GH × 4) and 16 GB RAM. We experimentally find that it can analyze the two datasets considered within 2 seconds (see Additional file 1). Compared to the time complexity reported in [12], our method is more efficient.

Unlike studies that de-noise the interactions obtained from high-throughput experiments through predicting missing interactions and identifying spurious interactions [11, 46, 47], this study attempts to distinguish direct physical interactions and indirect co-complex interactions in AP-MS data. The de-noising methods basically assume that two proteins sharing many common neighbors or having short distance with some measures are likely to interact with each other. They prefer to assign high scores to co-complex interactions and low scores to inter-complex interactions [46]. However, inter-complex interactions may be direct and intra-complex interactions may be indirect [29]. Therefore, they cannot be applied to discriminate direct and indirect interactions. In previous studies, indirect secondary data, such as coexpression, functional annotation and phenotypic profile, are used to evaluate the performance of de-noising methods. Here we do not consider these secondary data since they often correlate with functional interactions [32] and do not have distinguishable distributions between direct and indirect interactions [5, 9]. As an alternative, we evaluate our method using known binary interactions in public databases and other three reference sets that provide less direct evidence for physical interactions [13, 33].

Our method may be improved in the following aspects. We only use the topology of the network to identify direct interactions. The performance depends on the topological structure of network and confidence scores of interactions under consideration. However, AP-MS data have a high level of noise and are incomplete [11]. Therefore, the predictions of our method may be limited in accuracy. One possible way to ameliorate this is to incorporate other genomic features (e.g, sequence, domain and three dimensional structure) that can provide evidence for physical interactions. In addition, we test our model on Saccharomyces cerevisiae since it is well studied and the comprehensive AP-MS data and reference sets are available. Recently, experimental data for other organisms (e.g., Drosophila melanogaster [48] and Homo sapiens [49–51]) become available, we will apply our model on these species to enrich the binary protein-protein interaction landscape.

## Conclusions

In this study, a new scoring method is developed to identify binary interactions from AP-MS data. Experiment results on two yeast datasets show the competitive performance of our method with respect to four types of reference sets. This study provide new insights for understanding and analyzing AP-MS data.

## Additional file

Additional file 1
**Supplementary figures and text.** This file provides the supplementary figures (Figures S1-S7) that illustrate the comparative performances and the supplementary text which presents the parameter estimation method, convergence analysis, computational complexity analysis and comparative experiments with other regularization methods. (PDF 406 KB)

## References

[1] Mitra K, Carvunis AR, Ramesh SK, Ideker T (2013). Integrative approaches for finding modular structure in biological networks. Nat Rev Genet.

[2] Wang X, Wei X, Thijssen B, Das J, Lipkin SM, Yu H (2012). Three-dimensional reconstruction of protein networks provides insight into human genetic disease. Nat Biotechnol.

[3] Young K (1998). Yeast two-hybrid: so many interactions,(in) so little time. Biol Reprod.

[4] Ito T, Chiba T, Ozawa R, Yoshida M, Hattori M, Sakaki Y (2001). A comprehensive two-hybrid analysis to explore the yeast protein interactome. Proc Natl Acad Sci USA.

[5] Rajagopala SV, Sikorski P, Kumar A, Mosca R, Vlasblom J (2014). The binary protein-protein interaction landscape of escherichia coli. Nat Biotechnol.

[6] Gavin AC, Aloy P, Grandi P, Krause R, Boesche M, Marzioch M (2006). Proteome survey reveals modularity of the yeast cell machinery. Nature.

[7] Krogan NJ, Cagney G, Yu H, Zhong G, Guo X, Ignatchenko A (2006). Global landscape of protein complexes in the yeast Saccharomyces cerevisiae. Nature.

[8] Ewing RM, Chu P, Elisma F, Li H, Taylor P, Climie S (2007). Large-scale mapping of human protein–protein interactions by mass spectrometry. Mol Syst Biol.

[9] Yu H, Braun P, Yıldırım MA, Lemmens I, Venkatesan K, Sahalie J (2008). High-quality binary protein interaction map of the yeast interactome network. Science.

[10] Deane CM, Salwiński Ł, Xenarios I, Eisenberg D (2002). Protein interactions two methods for assessment of the reliability of high throughput observations. Mol Cell Proteomics.

[11] Kuchaiev O, Rašajski M, Higham DJ, Pržulj N (2009). Geometric de-noising of protein-protein interaction networks. PLoS Comput Biol.

[12] Kim E, Sabharwal A, Vetta A, Blanchette M (2010). Predicting direct protein interactions from affinity purification mass spectrometry data. Algorithms Mol Biol.

[13] Schelhorn SE, Mestre J, Albrecht M, Zotenko E (2011). Inferring physical protein contacts from large-scale purification data of protein complexes. Mol Cell Proteomics.

[14] Teng B, Zhao C, Liu X, He Z (2015). Network inference from ap-ms data: computational challenges and solutions. Brief Bioinform.

[15] Collins SR, Kemmeren P, Zhao XC, Greenblatt JF, Spencer F, Holstege FC (2007). Toward a comprehensive atlas of the physical interactome of Saccharomyces cerevisiae. Mol Cell Proteomics.

[16] Friedel CC, Krumsiek J, Zimmer R (2009). Bootstrapping the interactome: unsupervised identification of protein complexes in yeast. J Comput Biol.

[17] Xie Z, Kwoh CK, Li XL, Wu M (2011). Construction of co-complex score matrix for protein complex prediction from ap-ms data. Bioinformatics.

[18] Choi H, Larsen B, Lin ZY, Breitkreutz A, Mellacheruvu D, Fermin D (2011). Saint: probabilistic scoring of affinity purification-mass spectrometry data. Nat Methods.

[19] Pu S, Vlasblom J, Turinsky A, Marcon E, Phanse S, Trimble SS (2015). Extracting high confidence protein interactions from affinity purification data: At the crossroads. J Proteomics.

[20] Friedel CC, Zimmer R (2009). Identifying the topology of protein complexes from affinity purification assays. Bioinforma.

[21] Bader GD, Hogue CW (2002). Analyzing yeast protein–protein interaction data obtained from different sources. Nat Biotechnol.

[22] Saraç ÖS, Pancaldi V, Bähler J, Beyer A (2012). Topology of functional networks predicts physical binding of proteins. Bioinforma.

[23] Barzel B, Barabási AL (2013). Network link prediction by global silencing of indirect correlations. Nat Biotechnol.

[24] Feizi S, Marbach D, Médard M, Kellis M (2013). Network deconvolution as a general method to distinguish direct dependencies in networks. Nat Biotechnol.

[25] Hastie T, Tibshirani R, Friedman J (2009). The Elements of Statistical Learning: Data Mining, Inference, and Prediction.

[26] Robertson T, Wright F, Dykstra RL, Robertson T (1988). Order Restricted Statistical Inference.

[27] Efron B (1979). Bootstrap methods: another look at the jackknife. Ann Stat.

[28] Efron B, Tibshirani RJ (1994). An Introduction to the Bootstrap.

[29] Das J, Yu H (2012). Hint: High-quality protein interactomes and their applications in understanding human disease. BMC Syst Biol.

[30] Tarassov K, Messier V, Landry CR, Radinovic S, Molina MMS, Shames I (2008). An in vivo map of the yeast protein interactome. Science.

[31] Chatr-aryamontri A, Breitkreutz BJ, Heinicke S, Boucher L, Winter A, Stark C (2013). The biogrid interaction database: 2013 update. Nucleic Acids Res.

[32] Zhang QC, Petrey D, Deng L, Qiang L, Shi Y, Thu CA (2012). Structure-based prediction of protein-protein interactions on a genome-wide scale. Nature.

[33] Zhang QC, Petrey D, Garzón JI, Deng L, Honig B (2013). Preppi: a structure-informed database of protein–protein interactions. Nucleic Acids Res.

[34] Pu S, Wong J, Turner B, Cho E, Wodak SJ (2009). Up-to-date catalogues of yeast protein complexes. Nucleic Acids Res.

[35] Cherry JM, Adler C, Ball C, Chervitz SA, Dwight SS, Hester ET (1998). SGD: Saccharomyces genome database. Nucleic Acids Res.

[36] Nepusz T, Yu H, Paccanaro A (2012). Detecting overlapping protein complexes in protein-protein interaction networks. Nat Methods.

[37] Zhang XF, Dai DQ, Ou-Yang L, Wu MY (2012). Exploring overlapping functional units with various structure in protein interaction networks. PLoS ONE.

[38] Ashburner M, Ball CA, Blake JA, Botstein D, Butler H, Cherry JM (2000). Gene ontology: tool for the unification of biology. Nat Genet.

[39] Costanzo M, Baryshnikova A, Bellay J, Kim Y, Spear ED, Sevier CS (2010). The genetic landscape of a cell. Science.

[40] Lee DD, Seung HS (2001). Algorithms for Non-negative Matrix Factorization. Adv Neural Inf Process Syst, vol. 13.

[41] Fawcett T (2006). An introduction to roc analysis. Pattern Recogn Lett.

[42] Zhang XF, Dai DQ, Ou-Yang L, Yan H (2014). Detecting overlapping protein complexes based on a generative model with functional and topological properties. BMC Bioinforma.

[43] van Rijsbergen C (1979). Information Retrieval.

[44] *F*_2_ measure. https://en.wikipedia.org/wiki/F1_score. Access date 10 July 2015.

[45] Ben-Hur A, Noble W (2006). Choosing negative examples for the prediction of protein-protein interactions. BMC Bioinforma.

[46] Lei C, Ruan J (2013). A novel link prediction algorithm for reconstructing protein–protein interaction networks by topological similarity. Bioinforma.

[47] Zhu Y, Zhang XF, Dai DQ, Wu MY (2013). Identifying spurious interactions and predicting missing interactions in the protein-protein interaction networks via a generative network model. IEEE/ACM Trans Comput Biol Bioinform.

[48] Guruharsha K, Rual JF, Zhai B, Mintseris J, Vaidya P, Vaidya N (2011). A protein complex network of drosophila melanogaster. Cell.

[49] Sowa ME, Bennett EJ, Gygi SP, Harper JW (2009). Defining the human deubiquitinating enzyme interaction landscape. Cell.

[50] Behrends C, Sowa ME, Gygi SP, Harper JW (2010). Network organization of the human autophagy system. Nature.

[51] Marcon E, Ni Z, Pu S, Turinsky AL, Trimble SS, Olsen JB (2014). Human-chromatin-related protein interactions identify a demethylase complex required for chromosome segregation. Cell Rep.

