# Supplementary material for: Identifying binary protein-protein interactions from affinity purification mass spectrometry data
Source: BMC Genomics. 2015 Oct 5;16:745. doi: 10.1186/s12864-015-1944-z (PMC4595009; doi:10.1186/s12864-015-1944-z)
Supplement: Additional file 1 — Supplementary figures and text. This file provides the supplementary figures (Figures S1-S7) that illustrate the comparative performances and the supplementary text which presents the parameter estimation method, convergence analysis, computational complexity analysis and comparative experiments with other regularization methods. (PDF 406 KB) [file 12864_2015_1944_MOESM1_ESM.pdf]

Identifying binary protein-protein interactions from affinity purification mass spectrometry data: Additional file 1

Xiao-Fei Zhang, Le Ou-Yang, Xiaohua Hu and Dao-Qing Dai

Contents

1 Supplementary Figures **2**

2 Supplementary Text **6**

2.1 Model parameter estimation 6

2.2 Convergence analysis 6

2.3 Computational complexity analysis 6

2.4 Comparison with other regularization methods 7

1 Supplementary Figures

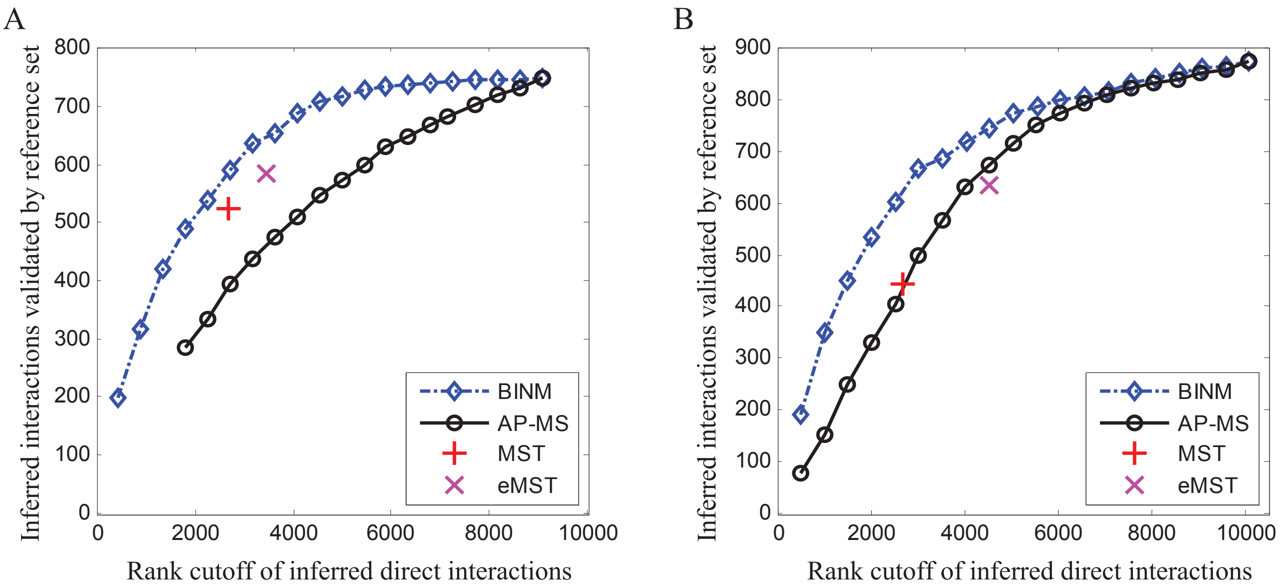

Figure S1: **Assessment of inferred direct interactions against the Y2H reference set.** Interactions are ranked by scores calculated using corresponding methods. Performance of all methods is measured by plotting the number of top-ranking inferred direct interactions of a method against the number of these interactions that are validated by the Y2H reference set. (A) Collins dataset, (B) Friedel dataset.

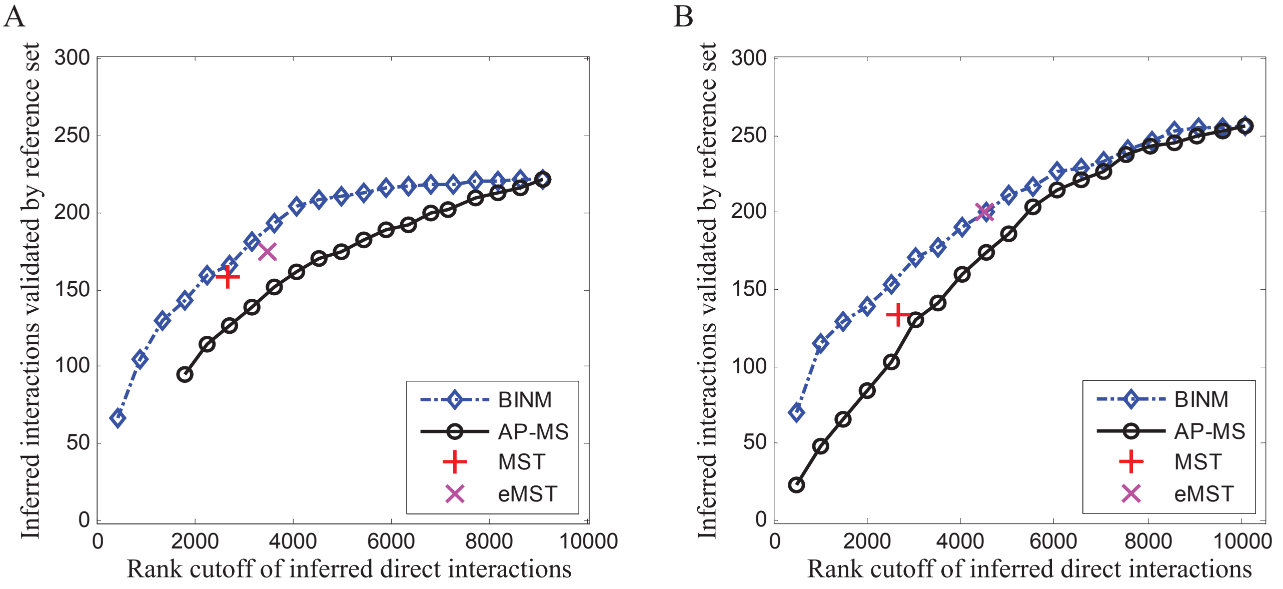

Figure S2: **Assessment of inferred direct interactions against the PCA reference set.** Interactions are ranked by scores calculated using corresponding methods. Performance of all methods is measured by plotting the number of top-ranking inferred direct interactions of a method against the number of these interactions that are validated by the PCA reference set. (A) Collins dataset, (B) Friedel dataset.

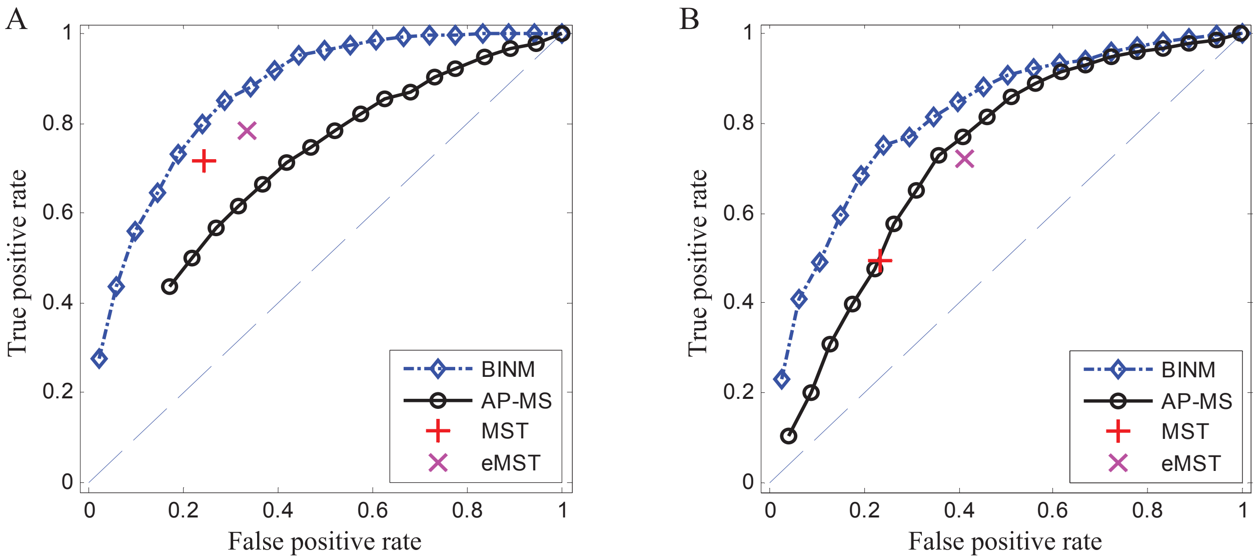

Figure S3: **ROC curves for direct (versus indirect) interaction classification with respect to HINT reference sets.** (A) Collins dataset, (B) Friedel dataset.

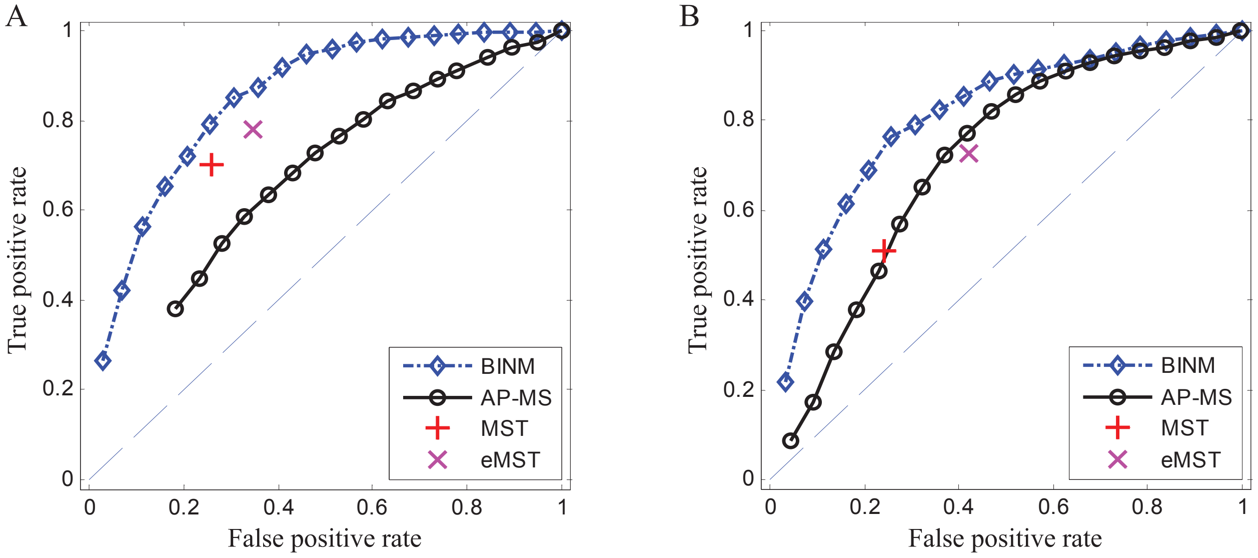

Figure S4: **ROC curves for direct (versus indirect) interaction classification with respect to Y2H reference sets.** (A) Collins dataset, (B) Friedel dataset.

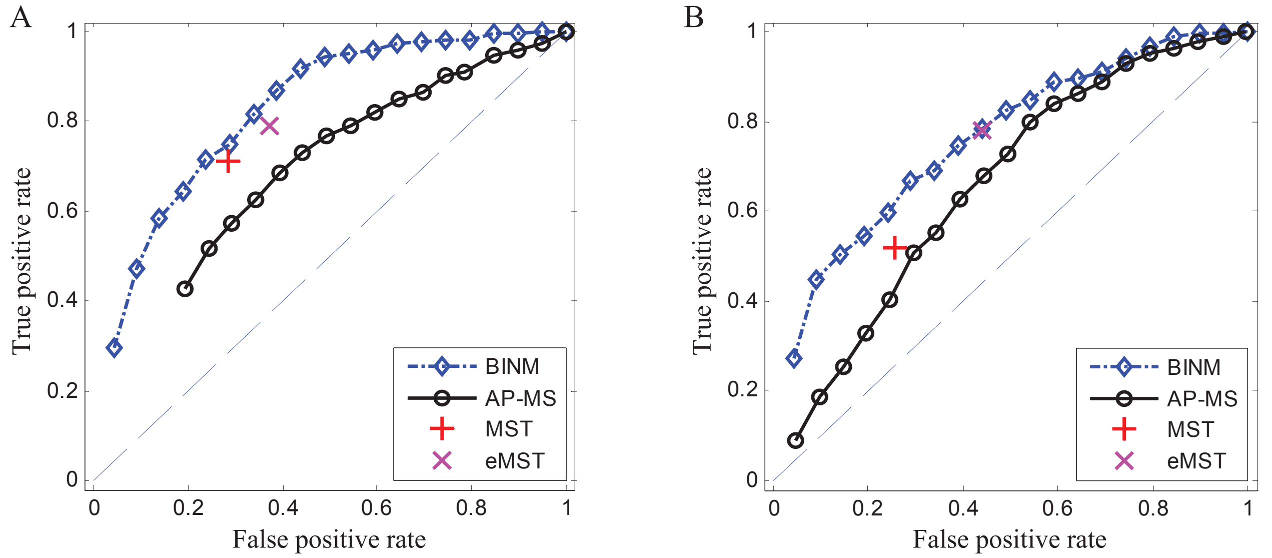

Figure S5: **ROC curves for direct (versus indirect) interaction classification with respect to PCA reference sets.** (A) Collins dataset, (B) Friedel dataset.

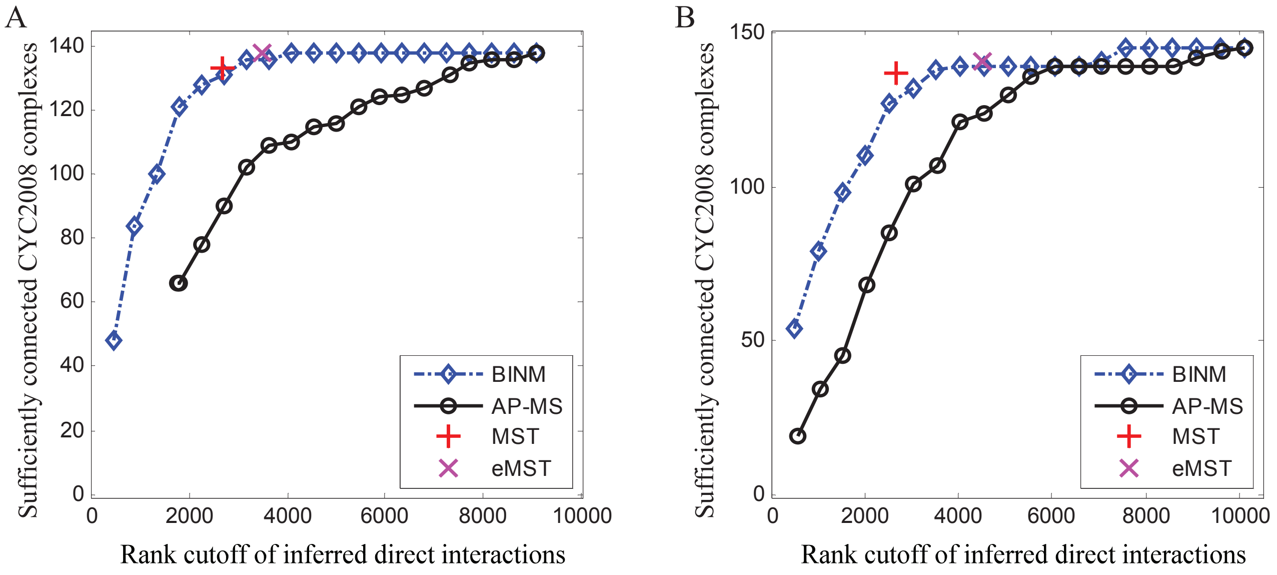

Figure S6: **Assessment of inferred direct interactions using the CYC2008 complexes.** Interactions are ranked by scores calculated using corresponding methods. Performance of all methods is measured by plotting the number of top-ranking inferred direct interactions of a method against the number of complexes that are sufficiently connected by these interactions. A complex is considered to be sufficiently connected by a set of physical interactions if the physical interactions reduce the number of connected components within the complex to less than 50% compared with the unconnected complex. (A) Collins dataset, (B) Friedel dataset.

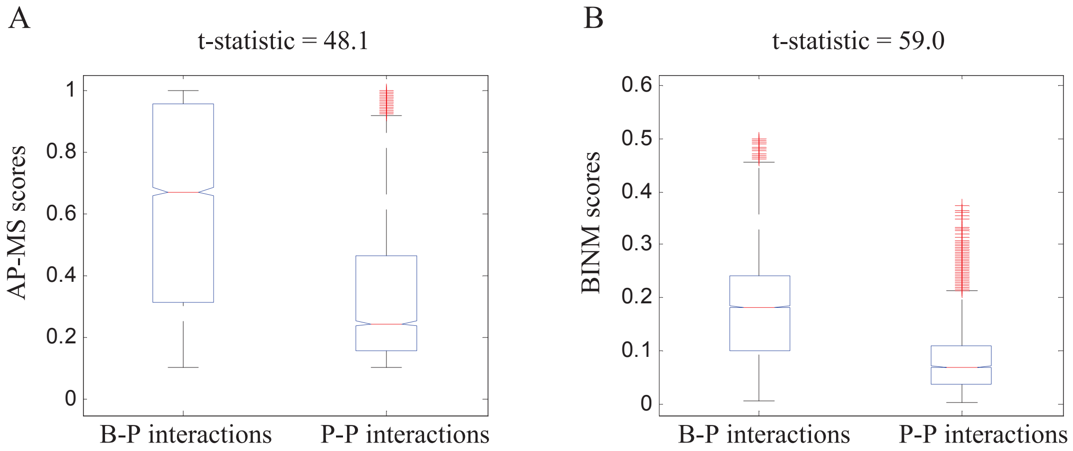

Figure S7: **Score distributions of different types of interactions on the Friedel dataset.** The score distributions are represented by box plots (line = median). We use Student's t-test to test difference between scores of bait-prey (B-P) interactions and prey-prey (P-P) interactions, and t-statistics is presented in the figure. (A) AP-MS score distributions, (B) BINM score distributions.

2 Supplementary Text

2.1 Model parameter estimation

The objective function of the proposed binary interaction network model (BINM) is

$$\min_{W_{dir}\geq 0} \quad \|\mathcal{D}\left(W_{obs} - \left(W_{dir} + W_{dir}^2\right)\right)\|_F^2 + \lambda\|W_{dir}\|_F^2, \tag{1}$$

where $W_{dir} \geq 0$ means each element $w_{ij}^{dir} \geq 0$ and $\mathcal{D}(\cdot)$ sets the diagonal terms of a matrix to zero.

We use the multiplicative updating rule [1] to solve this nonnegative constrained optimization problem. Let ω_{ij} be the Lagrange multipliers for constraints $w_{ij}^{dir} \geq 0$, and $\Omega = (\omega_{ij})$. The Lagrange function $\mathcal{L}$ is

$$\mathcal{L}\left(W_{dir}, \Omega\right) \ = \ \|\mathcal{D}\left(W_{obs} - \left(W_{dir} + W_{dir}^2\right)\right)\|_F^2 + \lambda\|W_{dir}\|_F^2 + tr(\Omega W_{dir}^T), \tag{2}$$

where $tr(\cdot)$ denotes the trace of a matrix. The partial derivatives of $\mathcal{L}$ with respect to W_{dir} is:

$$\frac{\partial\mathcal{L}}{\partial W_{dir}} \ = \ -2W_{obs} - 2{W_{dir}}^T W_{obs} - 2W_{obs}{W_{dir}}^T + 2\hat{W}_{obs} + 2{W_{dir}}^T\hat{W}_{obs} + 2\hat{W}_{obs}{W_{dir}}^T + 2\lambda W_{dir} + \Omega, \tag{3}$$

where $\hat{W}_{obs} = \mathcal{D}\left(W_{dir} + W_{dir}^2\right)$. Since the estimators of W_{dir} need to satisfy $\frac{\partial\mathcal{L}}{\partial W_{dir}} \ = \ 0$, we can get

$$\Omega \ = \ 2W_{obs} + 2{W_{dir}}^T W_{obs} + 2W_{obs}{W_{dir}}^T - 2\hat{W}_{obs} - 2{W_{dir}}^T\hat{W}_{obs} - 2\hat{W}_{obs}{W_{dir}}^T - 2\lambda W_{dir}. \tag{4}$$

By the Karush-Kuhn-Tucker (KKT) conditions [2], $w_{ij}^{dir}\omega_{ij} = 0$, we get the following equations for w_{ij}^{dir}:

$$w_{ij}^{dir}\left(W_{obs} + {W_{dir}}^T W_{obs} + W_{obs}{W_{dir}}^T - \hat{W}_{obs} - {W_{dir}}^T\hat{W}_{obs} - \hat{W}_{obs}{W_{dir}}^T - \lambda W_{dir}\right)_{ij} = 0. \tag{5}$$

This leads to the following update rule for w_{ij}^{dir},

$$w_{ij}^{dir} \leftarrow w_{ij}^{dir} \cdot \left(\frac{W_{obs} + {W_{dir}}^T W_{obs} + W_{obs}{W_{dir}}^T}{\hat{W}_{obs} + {W_{dir}}^T\hat{W}_{obs} + \hat{W}_{obs}{W_{dir}}^T + \lambda W_{dir}}\right)_{ij}^{\cdot\frac{1}{4}}. \tag{6}$$

For the sake of convenience, we rewrite the updating formulae in matrix form,

$$W_{dir} \leftarrow W_{dir} \cdot \left(\frac{W_{obs} + {W_{dir}}^T W_{obs} + W_{obs}{W_{dir}}^T}{\hat{W}_{obs} + {W_{dir}}^T\hat{W}_{obs} + \hat{W}_{obs}{W_{dir}}^T + \lambda W_{dir}}\right)^{\cdot\frac{1}{4}}. \tag{7}$$

Here matrix operation $X \cdot Y$ represents element-by-element multiplication; $\frac{X}{Y}$ represents element-by-element division; and $X^{\cdot\frac{1}{4}}$ represents element power.

2.2 Convergence analysis

We have developed an iterative algorithm to solve the optimization problem of BINM based on multiplicative updating rule. It is known that the multiplicative updating rule is a special case of gradient descent methods with an automatic step parameter selection for guaranteeing the nonnegativity of parameters [3]. It may therefore be able to prove that the objective function of our model is nonincreasing under the update and that the iterative algorithm is guaranteed to find at least locally optimal solutions by constructing an auxiliary function similar to that used in [3, 4]. Instead of proving this in theory, we validate the convergence experimentally. From Figigure S8, we observe that the objective function decreases under the updating process, which demonstrates the convergence of our algorithm.

2.3 Computational complexity analysis

We now analyze the computational complexity in Equation (7). Since matrices W_{obs} and W_{dir} are sparse, computing W_{dir}^2, ${W_{dir}}^T W_{obs}$, $W_{obs}{W_{dir}}^T$, ${W_{dir}}^T\hat{W}_{obs}$, and $\hat{W}_{obs}{W_{dir}}^T$ take $O(nE)$ times in the worst case, where n is the number of proteins and E is the number of interactions. Consequently, the time cost to update W_{obs} once is $O(nE)$, and hence the time complexity of our method is $O(nTE)$, where T is the iteration number for convergence. In real world situations, the co-complex interaction networks are extremely sparse. Therefore, the overall cost might be reduced in practice. In the experiments, we implement the algorithm using Matlab in a workstation with Intel 4 CPU (3.40 GH $\times$ 4) and 16 GB RAM. As can be seen from Table S1, each update costs at most 0.07 seconds and the entire estimation takes less than 2 seconds. Therefore, our method is efficient and scale well with the size of the network analyzed.

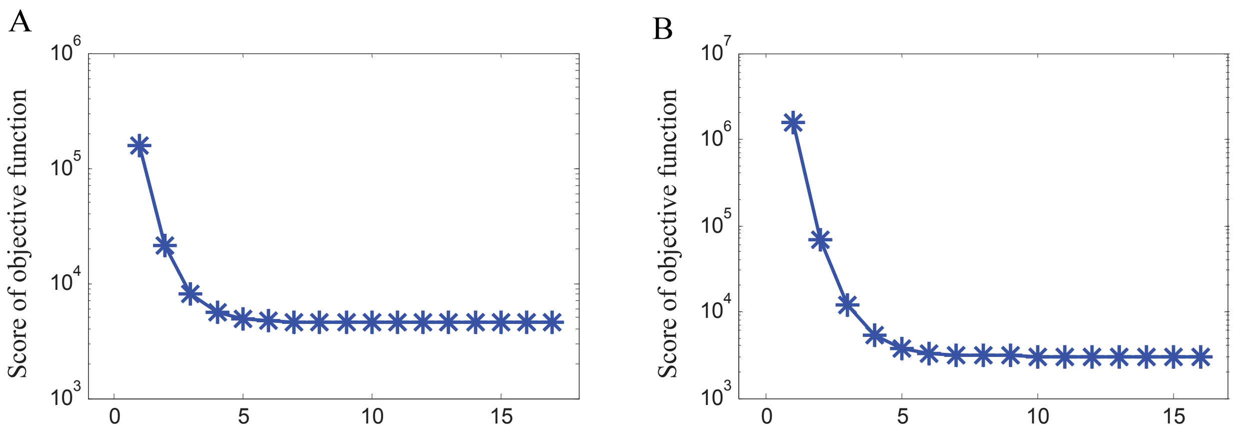

Figure S8: Convergence analysis of parameter estimation algorithm. For each figure, the x-axis denotes the number of iterations, and the y-axis denotes the value of the objective function in logarithm scale. (A) Collins dataset, (B) Friedel dataset.

Table S1: Time cost (second) of BINM for estimating model parameters

Dataset	Per update	Entire estimation
Collins	0.068	1.15
Friedel	0.062	0.94

2.4 Comparison with other regularization methods

In our model (Equation 1), following the method of ridge regression [5], we use an ℓ_2-norm (Frobenius norm) regularization term $\|W_{dir}\|_F^2$ to avoid overfitting. In fact, since it would except that a co-complex interaction network would be sparse, an ℓ_1-norm regularization term $\|W_{dir}\|_1$, which is similar to lasso [5], may be better suited to identify direct physical interactions. To test the effect of ℓ_1-norm penalty, we change our model as follows:

$$\min_{W_{dir}\geq 0} \quad \|\mathcal{D}\left(W_{obs} - \left(W_{dir} + W_{dir}^2\right)\right)\|_F^2 + \lambda \|W_{dir}\|_1, \tag{8}$$

where $\|W_{dir}\|_1 = \sum_{i=1}^n \sum_{j=1}^n w_{ij}^{dir}$. Here we use a ℓ_1-norm penalty to replace the ℓ_2-norm penalty. According to the multiplicative update rule, we obtain the following updating formula:

$$W_{dir} \leftarrow W_{dir} \cdot \left(\frac{W_{obs} + {W_{dir}}^T W_{obs} + W_{obs} {W_{dir}}^T}{\hat{W}_{obs} + {W_{dir}}^T \hat{W}_{obs} + \hat{W}_{obs} {W_{dir}}^T + \frac{\lambda}{2}} \right)^{\cdot \frac{1}{4}}, \tag{9}$$

where $\hat{W}_{obs}$ is computed using $\hat{W}_{obs} = \mathcal{D}\left(W_{dir} + W_{dir}^2\right)$. Then the estimator of W_{dir} can be obtained according to this update rule.

We compare the performances of ℓ_1-norm and ℓ_2-norm penalties. We run the BINM model with these two types of penalties on the two AP-MS datasets (Collins and Friedel) with different values of λ $\left(\lambda \in \{2^{-4}, 2^{-2}, \cdots, 2^2\}\right)$, and evaluate the performance with respect to the HINT reference set. The performance is measured by the area under the ROC curve (AUC). From Figure S9, we observe that when λ is small ($\lambda \in [2^{-4}, 2^{-2}]$), there is no clear difference between the performances of these two penalties. This might be due to the fact that the penalties have a slight effect on the estimators of W_{dir} when λ is small. However, when λ is large ($\lambda \in [2^{-1}, 2^2]$), ℓ_2-norm penalty outperforms ℓ_1-norm penalty significantly. These results shows that ℓ_2-norm penalty is less sensitive to the choice of λ than ℓ_1-norm penalty. Therefore, we use the ℓ_2-norm penalty in the main text.

In fact, various regularization methods have been proposed in the field of machine learning in recent years, such as lasso [6], SCAD penalties [7], elastic net [8], adaptive lasso [9], reweighted ℓ_1 minimization [10]. In our study, we do not compare the ℓ_2-norm regularization method with these methods for the following two reasons: (1) as shown in Figure 2 in the main text and Figure S9, the performance of BINM is not very sensitive to regularization parameter (e.g., λ) and a small λ can produce competitive performance; (2) the main contribution of BINM is the first term that captures topological relationships between observed co-complex interactions and underlying direct physical interactions. Consequently, we just use a widely used regularization term to avoid the case of overfitting.

References

[1] Daniel D. Lee and H. Sebastian Seung. Algorithms for non-negative matrix factorization. In *Advances in neural information processing systems*, volume 13, pages 556–562, 2001.

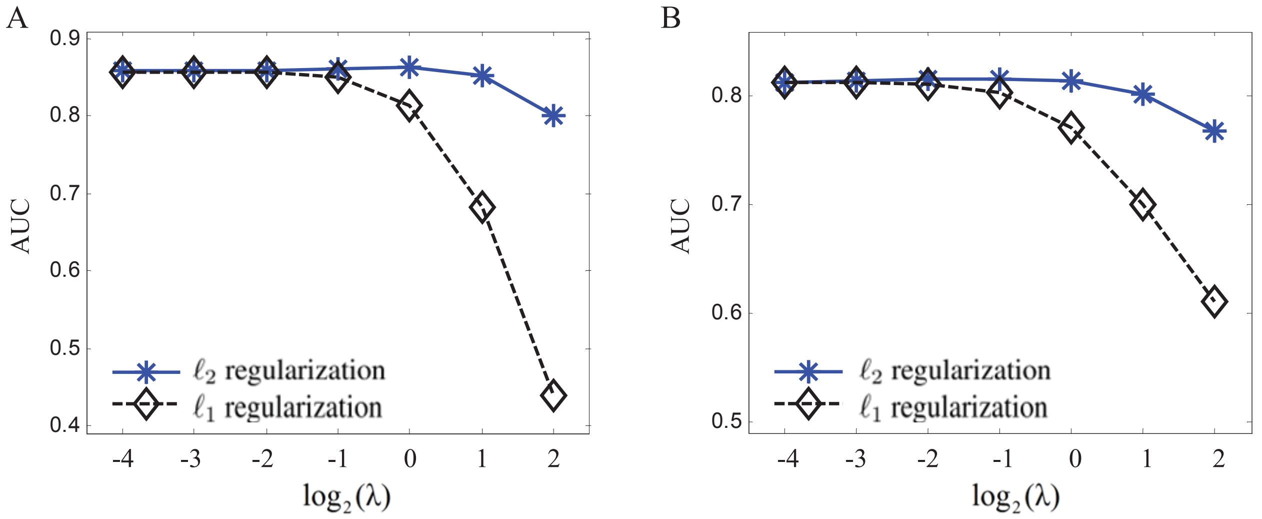

Figure S9: **Comparative performances of ℓ_1-norm and ℓ_2-norm penalties.** The x-axis denotes the value of $\log_2 \lambda$; the y-axis denotes the value of AUC score. (A) Collins dataset, (B) Friedel dataset.

[2] H.W. Kuhn and A.W. Tucker. Nonlinear programming. In *Proceedings of the second Berkeley symposium on mathematical statistics and probability*, volume 1, pages 481–492. California, 1951.

[3] Deng Cai, Xiaofei He, Jiawei Han, and Thomas S Huang. Graph regularized nonnegative matrix factorization for data representation. *IEEE Transactions on Pattern Analysis and Machine Intelligence*, 33(8):1548–1560, 2011.

[4] D Seung. Algorithms for non-negative matrix factorization. *Advances in neural information processing systems*, 13:556–562, 2001.

[5] Trevor Hastie, Robert Tibshirani, and Jerome Friedman. *The Elements of Statistical Learning: Data Mining, Inference, and Prediction*. Springer, Berlin, 2009.

[6] Robert Tibshirani. Regression shrinkage and selection via the lasso. *Journal of the Royal Statistical Society. Series B (Methodological)*, pages 267–288, 1996.

[7] Jianqing Fan and Runze Li. Variable selection via nonconcave penalized likelihood and its oracle properties. *Journal of the American Statistical Association*, 96(456):1348–1360, 2001.

[8] Hui Zou and Trevor Hastie. Regularization and variable selection via the elastic net. *Journal of the Royal Statistical Society: Series B (Statistical Methodology)*, 67(2):301–320, 2005.

[9] Hui Zou. The adaptive lasso and its oracle properties. *Journal of the American Statistical Association*, 101(476):1418–1429, 2006.

[10] Emmanuel J Candes, Michael B Wakin, and Stephen P Boyd. Enhancing sparsity by reweighted ℓ_1 minimization. *Journal of Fourier Analysis and Applications*, 14(5-6):877–905, 2008.
